# Differential expression and localization of TIMP-1 and TIMP-4 in human gliomas

**DOI:** 10.1054/bjoc.2001.1854

**Published:** 2001-07

**Authors:** L L Groft, H Muzik, N B Rewcastle, R N Johnston, V Knäuper, M A Lafleur, P A Forsyth, D R Edwards

**Affiliations:** Departments of Biochemistry and Molecular Biology and; Clinical Neurosciences, The University of Calgary, 3330 Hospital Drive NW, Calgary, Alberta, T2N 4N1, Canada; School of Biological Sciences, University of East Anglia, Norwich, NR4 7TJ, England; Departments of Oncology and Clinical Neurosciences, University of Calgary; Tom Baker Cancer Centre, Calgary, Alberta, Canada; Department of Pathology, Foothills Hospital, Calgary, Alberta, Canada

**Keywords:** gliomas, metalloproteinases, TIMPs, RT-PCR

## Abstract

Studies have suggested that an imbalance of matrix metalloproteinases (MMPs) and tissue inhibitors of metalloproteinases (TIMPs) may contribute to the malignant phenotype of gliomas. In this study, we have undertaken a detailed analysis of expression of the TIMP family in normal human brain and malignant gliomas at both the mRNA and protein level. Reverse transcription-PCR (RT-PCR) analyses of total RNA from surgical tumour specimens revealed unique expression patterns for the 4 members of the TIMP family, with TIMP-1 and -4 showing positive and negative correlations, respectively, with glioma malignancy. By RT-PCR, TIMP-2 and TIMP-3 expression did not change with tumour grade. In situ hybridization localized TIMP-1 to glial tumour cells and also to the surrounding tumour vasculature. TIMP-4 transcripts were predominantly localized to tumour cells, though minor expression was found in vessels. Recombinant TIMP-4 reduced invasion of U251 glioma cells through Matrigel, and U87 clones overexpressing TIMP-4 showed reduced invasive capacity in vitro. TIMP-4, but not TIMP-1, blocked Membrane Type-1-MMP-mediated progelatinase-A (MMP-2) activation in human umbilical vein endothelial cells. The differential expression and localization of individual TIMPs may contribute to the pathophysiology of human malignant gliomas, particularly with regard to tumour vascularization. © 2001 Cancer Research Campaign http://www.bjcancer.com


*British Journal of Cancer* (2001) **85**(1), 55–63
© 2001 Cancer Research Campaign
doi: 10.1054/ bjoc.2001.1854, available online at http://www.idealibrary.com on IDEAL®

http://www.bjcancer.com

### Differential expression and localization of TIMP-1 and TIMP-4 in human gliomas

**LL Groft[1], H Muzik[1], NB Rewcastle[2,6], RN Johnston[1], V Knäuper[3], MA Lafleur[3], PA Forsyth[2,4,5] and DR Edwards[3]**

Departments of [1]Biochemistry and Molecular Biology and [2]Clinical Neurosciences, The University of Calgary, 3330 Hospital Drive NW, Calgary, Alberta, T2N 4N1, Canada; [3]School of Biological Sciences, University of East Anglia, Norwich, NR4 7TJ, England; [4]Departments of Oncology and Clinical Neurosciences, University of Calgary; [5]Tom Baker Cancer Centre, Calgary, Alberta, Canada; [6]Department of Pathology, Foothills Hospital, Calgary, Alberta, Canada

**Summary** Studies have suggested that an imbalance of matrix metalloproteinases (MMPs) and tissue inhibitors of metalloproteinases (TIMPs) may contribute to the malignant phenotype of gliomas. In this study, we have undertaken a detailed analysis of expression of the TIMP family in normal human brain and malignant gliomas at both the mRNA and protein level. Reverse transcription-PCR (RT-PCR) analyses of total RNA from surgical tumour specimens revealed unique expression patterns for the 4 members of the TIMP family, with TIMP-1 and -4 showing positive and negative correlations, respectively, with glioma malignancy. By RT-PCR, TIMP-2 and TIMP-3 expression did not change with tumour grade. In situ hybridization localized TIMP-1 to glial tumour cells and also to the surrounding tumour vasculature. TIMP-4 transcripts were predominantly localized to tumour cells, though minor expression was found in vessels. Recombinant TIMP-4 reduced invasion of U251 glioma cells through Matrigel, and U87 clones overexpressing TIMP-4 showed reduced invasive capacity in vitro. TIMP-4, but not TIMP-1, blocked Membrane Type-1-MMP-mediated progelatinase-A (MMP-2) activation in human umbilical vein endothelial cells. The differential expression and localization of individual TIMPs may contribute to the pathophysiology of human malignant gliomas, particularly with regard to tumour vascularization. © 2001 Cancer Research Campaign http://www.bjcancer.com

**Keywords**: gliomas; metalloproteinases; TIMPs; RT-PCR

Malignant gliomas are both highly vascular and invasive tumours; tumour cells commonly invade several centimetres beyond the main tumour mass at the time of diagnosis. These features render them surgically incurable and existing treatments only modestly prolong survival. Both invasion and neovascularization require extracellular matrix (ECM) breakdown and the subsequent migration of cells through the degraded structures (Liotta et al, 1991). Since ECM remodelling is the principal activity of a family of enzymes known as matrix metalloproteinases (MMPs), these enzymes have come under investigation for their contributions to the malignant phenotype of gliomas (Uhm et al, 1997; Yong et al, 1998; Price et al, 1999; Raithatha et al, 2000).

There are currently 23 known members of the MMP family which are subdivided according to their structures and substrate specificities into membrane-type matrix metalloproteinases (MT-MMPs), collagenases, stromelysins and gelatinases (Murphy and Knäuper, 1997; Nagase and Woessner, 1999). Controlled MMP activity is essential to numerous physiological processes such as wound healing and bone development. However, when MMP activity goes unchecked, excessive tissue destruction occurs, leading to a myriad of pathological states including tumour invasion and metastasis. The MMPs are tightly regulated at several levels, including transcriptional induction, activation of inactive zymogens, and inhibition by tissue inhibitors of metalloproteinases (TIMPs). An imbalance between MMP and TIMP activity may contribute to the malignant behaviour of many cancers (Chambers and Matrisian, 1997). In particular, Gelatinase-A (MMP-2) and the MT-MMPs that activate it on the cell surface have been linked with both tumour cell invasion and angiogenesis (Yamamoto et al, 1996; Hiraoka et al, 1998; Belien et al, 1999; Forsyth et al, 1999; Llano et al, 1999; Nakada et al, 1999; Price et al, 1999; Raithatha et al, 2000).

The TIMP family is comprised of 4 gene products (TIMPs 1–4) which inhibit secreted MMPs with roughly comparable potencies (Apte et al, 1995; Greene et al, 1996; Blavier et al, 1999). Individual TIMPs differ markedly in their pro-MMP interactions and gene regulatory mechanisms. For example, TIMP-1 specifically interacts with pro-gelatinase-B and is subject to tight control at the level of transcription (Phillips et al, 1999), whereas TIMP-2 binds pro-gelatinase A and shows constitutive gene expression (Blavier et al, 1999). Differences also exist with regard to biochemical properties: TIMP-3 is itself an ECM-associated protein but TIMP-1, -2 and -4 are freely diffusible (Leco et al, 1994). Moreover, TIMP-1 is ineffective as an inhibitor of MT1-MMP, whereas TIMP-2 and TIMP-3 are both functional in this regard (Will et al, 1996).

There is broad support for the notion that TIMPs in general act as 'brakes' on the malignant process. Thus, antisense-mediated down-regulation of TIMP-1 can induce tumorigenic and metastatic behaviour in mouse fibroblasts (Khokha et al, 1989) and exogenous or overexpressed TIMP-1 and TIMP-2 reduce tumour invasion and metastasis and in vivo (DeClerck et al, 1991, 1992). TIMP-3 down-regulation has been noted at the invasive edge of highly aggressive colorectal carcinomas (Powe et al, 1997), and overexpressed TIMP-4 resulted in decreased invasive capacity of breast carcinoma cells (Wang et al, 1997).

This classical notion of TIMPs in tumour biology is rather narrow, however, and there is a growing appreciation that these

*Received 20 December 2000*
*Revised 5 March 2001*
*Accepted 20 March 2001*

*Correspondence to:* DR Edwards proteins may affect a broad spectrum of cellular behaviours. For example, TIMP-1 stimulates the proliferation of erythroid precursors (Gasson et al, 1985), and both TIMP-1 and TIMP-2 can positively influence the proliferation of numerous cell types (Hayakawa et al 1992, 1994; Wingfield et al, 1999). Additionally, TIMP-2 inhibits in vitro proliferation of human microvascular endothelial cells stimulated with bFGF (Murphy et al, 1993), and TIMP-3 promotes apoptosis (Ahonen et al, 1998; Baker et al, 1998), possibly through stabilization of TNF alpha receptors (Smith et al, 1997). High TIMP-1 levels have been linked with increased malignancy in human lymphomas (Kossakowska et al, 1991; Stetler-Stevenson et al, 1997) and colorectal cancer (Zeng et al, 1995; Holten-Anderson et al, 1999). These studies suggest that TIMPs have a variety of functions beyond MMP inhibition, though their precise role in other cellular processes remains unclear.

There is a wealth of evidence showing that MMPs are overexpressed in malignant gliomas but studies of TIMPs in gliomas give conflicting results. Reduced expression of TIMP-1 and -2 with increasing glioma grade has been reported, suggesting that a lack of inhibitor expression may contribute to a more aggressive glioma phenotype (Mohanam et al, 1995; Kachra et al, 1999). However others report an upregulation of TIMP-1 or -2 expression in malignant tumours (Nakano et al, 1995; Saxena et al, 1995; Lampert et al, 1998). No studies have described the expression of TIMP-4 in human brain tumours.

To gain further insight into how TIMPs might be involved in glioma pathophysiology, we examined the mRNA and protein expression of TIMPs 1–4 in human normal brain and glioma surgical specimens. We found that TIMPs -1 and -4 expression correlated with glioma malignancy in a positive and negative manner respectively. Localization studies showed different cellular distribution patterns for these 2 genes, with TIMP-1 transcripts strongly localized to vascular structures, while both TIMP-1 and -4 localized to tumour cells. Our results suggest that deregulated expression of TIMP-1 and -4 may contribute to glioma malignancy, and point to unique roles for these proteins during glioma tumorigenesis.

### METHODS

### Tissue collection

Procedures were performed under a general anaesthetic. Induction consisted of a combination of pentothal, fentanyl, and vecuronium with maintenance achieved using isoflurane and intermittent fentanyl as needed. During the craniotomy, mannitol (1 g $kg^{-1}$) was administered for cerebral decompression. Tumour specimens were obtained prior to thermal coagulation and placed immediately in liquid nitrogen and stored at –80˚C in the Neuro-specimen Tumour Bank. The study has been approved by the ethics board of the Faculty of Medicine, University of Calgary. All patients gave signed, informed consent for their tissue to be used. The following non-irradiated, newly diagnosed tissues were studied from the tumour bank: 19 glioblastoma multiforme (GBMs), 7 anaplastic astrocytomas (AA), 5 malignant oligodendrogliomas (MO), 8 low-grade gliomas (LG): these were compared to 4 controls (2 from frozen tissue from surgery unconnected with glioma and 2 obtained at autopsy).

### Cell culture

U87 and U251 cell lines were obtained from ATCC (American Type Culture Collection, Rockville, MD, USA). These cells were grown in Dulbecco's Modified Eagle's Medium-Ham's F12 (DMEM-F12) containing 10% (vol/vol) fetal calf serum. Cells were passaged after reaching approximately 80% confluency, harvested by trypsin treatment and replated in DMEM-F12/10% FCS.

Primary HUVECs were obtained from TCS Biologicals (Buckingham, UK) and grown on type I collagen (60 µg $ml^{-1}$) coated tissue culture plastic, in medium supplied by the manufacturer. For analysis of gelatinase levels in conditioned media, HUVECs were seeded on type I collagen (60 µg $ml^{-1}$) coated 24 well plates at a density of $1 \times 10^5$ cells per well. The following day, cells were washed twice with serum-free medium and incubated in fresh serum-free medium with or without stimulation with $10^{-7}$ M phorbol myristoyl-13 acetate (PMA; Sigma-Aldrich, Poole, UK) for 24 hours. The conditioned medium was then collected, centrifuged at 7000 rpm for 10 minutes to remove any cells in suspension, and 50 mM Tris pH 8.0 was added and stored at –20˚C.

### RNA and protein preparation

Total RNA was extracted using the acid guanidinium isothiocyanate method. The final RNA concentrations were determined by absorption at 260 nm using a GeneQuant spectrophotometer (Pharmacia). Protein extraction involved homogenization of tissue in extraction buffer (0.5 M Tris-HCl, pH 7.6, containing 0.2 M NaCl, 10 mM $CaCl_2$, and 1% Triton X-100). The homogenate was centrifuged at 4˚C for 15 minutes at 15 000 ***g*** and supernatant was stored at –70˚C for Western blot and reverse zymography assays.

### Reverse transcription reactions

Each 20 µl cDNA synthesis reaction contained 1 µg of total RNA, 1 × PCR buffer (10 mM Tris-HCl, pH 9.0, 50 mM KCl, 1.5 mM $MgCl_2$), 1 mM of each deoxyribonucleotide triphosphate, 20 U placental ribonuclease inhibitor (RNAguard, Pharmacia), 160 units of MuLV-reverse transcriptase (Bethesda Research Laboratories) and 100 pmol of random hexamer oligodeoxynucleotides (Pharmacia). Reaction mixtures were pre-incubated 10 min at 21˚C prior to cDNA synthesis. Reverse transcription was carried out for 50 min at 42˚C and then heated to 95˚C for 5 min to terminate the reaction. Samples were stored at –20˚C until use.

### Polymerase chain reactions

Multiplex PCRs were performed in 50 µl reaction volumes. Each reaction contained 2 µl of RT reaction product, 1 × PCR buffer, 80 µM of each deoxynucleotide, and 20 pmol of each 5′ and 3′ primer pair (see Table 1). 2 units of Taq DNA polymerase (Gibco-BRL) were added to each tube during the first denaturation step ('hot start') and equal aliquots (20 pmol) of GAPDH primer sets were added at the appropriate cycle number by the primer-dropping method (Wong et al, 1994). The primer-dropping method for quantification of MMP and TIMP RNA levels has been described in detail (Wong et al, 2000). Each PCR cycle consisted of a heat denaturation step (94˚C for 1 min), a primer-annealing step at

© 2001 Cancer Research Campaign

**Table 1** RT-PCR primers and conditions for TIMP expression analysis

| Target gene | Primer sequence (5′–3′) | Target accession number | Position | Product size (bp) | PCR cycle No. |
|---|---|---|---|---|---|
| TIMP- 1 | AGCGCCCAGAGAGACACC<br>CCACTCCGGGCAGGATT | X03124.1 | 33–50<br>702–686 | 670 | 25–26 |
| TIMP-2 | GGCGTTTTGCAATGCAGATGTAG<br>CACAGGAGCCGTCACTTCTCTTG | NM 003255.1 | 378–400<br>874–852 | 497 | 30–32 |
| TIMP-3 | CTTCTGCAACTCCGACATCGTG<br>TGCCGGATGCAGGCGTAGTGTTT | NM 000362.1 | 389–410<br>847–825 | 459 | 22–23 |
| TIMP-4 | AATCTCCAGTGAGAAGGTAGTTCC<br>CGATGTCAACAAACTCCTTCCTGA | NM 003256.1 | 212–235<br>723–700 | 512 | 31–32 |
| GAPDH | CGGAGTCAACGGATTTGGTCGTAT<br>AGCCTTCTCCATGGTGGTGAAGAC | M33197 | 78–101<br>384–361 | 307 | 22–23 |

(55˚C for 30 s) and a polymerization step (72˚C for 1 min). Reactions were performed in a Temp-Tronic Thermal Cycler (Barnstead/Thermolyne). PCR products were electrophoresed through a 2% agarose gel containing 0.2 μg $ml^{-1}$ ethidium bromide. Band intensities were calculated by scanning densitometry (NIH imaging program), and TIMP to GAPDH ratios for each sample were plotted as a function of tumour grade. Results were expressed as the mean ± standard deviation.

### Western blotting

20 μg of protein from glioma cell line conditioned medium were mixed with 3× SDS gel-loading buffer (0.188 mM Tris-HCl (pH 6.8), 3% SDS (w/v), 0.0075% bromophenol blue, 30% glycerol and 3% β-mercaptoethanol) and separated on a 12% polyacrylamide gel. After transfer to a nitrocellulose membrane, blots were blocked in 5% nonfat milk overnight at RT, and then incubated with 1 ng $ml^{-1}$ rabbit anti-human TIMP-4 antibody (Chemicon). After incubation with peroxidase labelled secondary antibodies, signals were detected using an enhanced chemiluminescence detection system (Amersham). For cell fractionation studies, glioma cells were washed twice in ice-cold PBS without $Ca^{2+}$ and $Mg^{2+}$ and then suspended in 1 ml hypotonic lysis buffer (10 mM Tris-HCl (pH 8.0), 0.2 mM KCl, 1 mM PMSF and 10 μg $ml^{-1}$ aprotinin and leupeptin). Cells were lysed by dounce homogenization and samples were adjusted to a final concentration of 0.25 M sucrose, 1 mM EDTA, and nuclei and unlysed cells were removed by low speed centrifugation (10 min at 1000 ***g***). Membrane and cytosol fractions were prepared from the postnuclear supernatant by centrifugation at 100 000 ***g*** for 1 h at 4˚C. The supernatant (cytosol) was removed and the remaining pellet (membrane fraction) was solubilized in SDS sample buffer. Both cytosol and membrane fractions were then analysed on Western blots as above.

### Gelatin zymography

MMP activity in the conditioned medium of cultured HUVECs was analysed by substrate gel electrophoresis (zymography), using a 10% polyacrylamide gel co-polymerized with 1 mg $ml^{-1}$ gelatin (Sigma). Equal amounts of samples were mixed with 1% (w/v) SDS sample buffer under non-reducing conditions and loaded onto the gel (resolving gel: 17.5 cm × 10.5 cm). Gels were run using a V15–17 GibcoBRL vertical gel electrophoresis apparatus, at 20 mA until the bromophenol blue dye traversed into the resolving gel. The current was then increased to 30 mA and the gels were electrophoresed until 1 hour after the bromophenol blue dye had run off the gel. The gels were then washed in 50 mM Tris-HCl, 5 mM $CaCl_2$ (pH 8.0) and 2.5% (v/v) Triton X-100 overnight and then incubated in 50 mM Tris-HCl, 5 mM $CaCl_2$ (pH 7.5) for 18 hours at 37˚C. Gels were stained with Coomassie blue and destained in 10% acetic acid and 10% isopropanol. Gelatinolytic activity appears as a clear band on a blue background.

### cRNA probe preparation

**TIMP-1:** A 185 bp HindIII/BamHI fragment of human TIMP-1 in pBluescript II $KS^-$ was used to generate antisense and sense riboprobes using T3 polymerase/Bam HI and T7 polymerase/Xho I digested plasmid respectively. **TIMP-4:** A 400 bp HindIII/BamHI fragment of murine TIMP-4 in pBluescript II $KS^-$ (Leco et al, 1997) was used to generate antisense and sense riboprobes using T3 polymerase/Bam HI and T7 polymerase/Hind III digested plasmid respectively. Riboprobes were prepared and labelled with digoxygenin (DIG)-labelled-UTP (Roche, Laval, Quebec, Canada) following the manufacturer's instructions.

### In situ hybridization

Tissue sections were deparaffinized using xylene and rehydrated through a graded ethanol series (100%, 95%, 80%, 70% respectively). After proteinase K treatment at 37˚C for 25 min and acetylation using a solution of 0.5% acetic anhyride in 0.1 M triethanolamine, pH 8.0, tissues were prehybridized for 2 hours at 50˚C (50% formamide, 5 × SSPE, 1 × Denhardt's solution) and 20 ng of probe (diluted in prehybridization buffer with 8 μg $ml^{-1}$ *E. coli* tRNA) was added. Sections were incubated at 60˚C overnight. Sections were washed in 2 X SSC and were incubated in 20 mg $ml^{-1}$ RNAse A at 37˚C for 45 min. After washing in a graded SSC series (2X, 1X, 0.5X, and 0.1 X (60˚C)) sections were incubated in blocking buffer containing lamb serum for 2 hours. Anti-DIG antibody was added at a concentration of 3/500 and slides were left for 4 hours at room temperature. NBT/BCIP chromogens were applied to sections and colour was developed in the dark. When the desired intensity was reached, the colour reaction was terminated by placing sections in 20 mM Tris-HCl pH 7.5, 10mM EDTA. Sections were mounted with Kaiser's medium. Photographs were taken using Kodak Royal Gold 35 MM film under a Ziess photomicroscope II under bright field illumination.

### Transfections

Mouse TIMP-4 cDNA (675 base pairs; Leco et al, 1997) was cloned into the EcoRI site of the pBabe-puro. TIMP-4 containing

© 2001 Cancer Research Campaign plasmids were introduced into U87 cells using the Calcium Phosphate Transfection Kit (Sigma) following the manufacturer's instructions, and cells were grown in DMEM F12/10% FCS selection medium with 20 μg $ml^{-1}$ puromycin. Cloning rings were used to isolate viable clones and functional expression of the TIMP-4 insert was confirmed using reverse zymography and Western blots.

### Invasion assay

Matrigel was thawed at 4˚C on ice overnight. Using cooled pipette tips, matrigel (5 μl) was mixed with 0.5% FBS (25 μl) and spread evenly in wells of a 24-well Boyden chamber containing 8 μm pore polycarbonate filters. Matrigel was allowed to solidify for 30 min at 37˚C. Cells were then seeded at a density of $2.5 \times 10^5$ $ml^{-1}$ into each chamber and covered with 200 μl of media (0.5% FBS) containing the desired concentration of inhibitor. Chambers were set into 700 μl of fibroblast media (ensuring no bubbles were caught under the chamber) and incubated for 24 h at 37˚C. Chambers were rinsed in PBS and cells were scraped off the top of the membrane using a cotton-tipped swab. Cells were fixed to the bottom of the chamber using cold methanol for 2 min and then stained in haematoxylin before mounting on slides with Kaiser's mounting media.

### RESULTS

### Analysis of TIMP gene expression in gliomas

To determine the relationship between TIMP expression and glioma aggressiveness, specimens from normal brain and tumour were analysed using RT-PCR. Total RNA was extracted from surgically obtained specimens, and each sample was amplified by PCR in 3 separate assays to ensure consistent results. GAPDH was used as an internal control in individual PCR reactions to monitor input levels of cDNA.

A representative profile for each of the 4 TIMPs is shown in Figure 1A and the combined quantified data for all of the tumour samples and normal brain specimens are shown in Figure 1B. When comparing the expression patterns of the 4 transcripts, only TIMPs-1 and -4 showed a correlation with glioma malignancy over the range of tumour grades. For TIMP-1, expression was barely detectable by RT-PCR in normal brain tissue and lower grade tumours, but increased dramatically for GBM tumours. In contrast, TIMP-4 mRNA expression showed a negative correlation with glioma grade, but was higher in tumours than normal brain. Relative to the GAPDH signal in each PCR reaction, the most intense TIMP-4 bands appeared for lower and middle grade lesions, while the ratio diminished for GBM specimens; some GBMs showed only a barely detectable TIMP-4 band. Whereas TIMP-1 and -4 expression varied with glioma malignancy, levels of TIMP-2 and TIMP-3 RNAs remained fairly constant over all tumour grades examined.

### Localization of TIMP-1 and TIMP-4 using in situ hybridization

To determine the cellular origin and distribution of TIMPs-1 and -4 we performed in situ hybridization on paraffin-embedded glioma and normal brain specimens. Adjacent sections were stained with haematoxylin and eosin to facilitate pathological interpretation. Strong localization of TIMP-1 transcript was seen in the cytoplasm of malignant cells, with little or no staining seen in the cells of the surrounding host stroma (Figure 2A). GBM sections also showed intense staining for TIMP-1 transcripts around vascular structures and within endothelial cells. This feature was noted in all high grade sections analysed, and was absent in lower grade astrocytomas. Normal brain showed low levels of expression only in neurons, and the TIMP-1 sense control showed no signal (data not shown).

Similar to TIMP-1, TIMP-4 transcripts were localized to malignant cells; however, in contrast to TIMP-1 there was very little signal from the TIMP-4 antisense probe in endothelial cells and adjacent perivascular cells (Figure 2B). TIMP-4 RNAs were also found in low-grade astrocytomas, and within the neurons of normal brain tissue, and TIMP-4 sense controls showed no signal (data not shown).

### Matrigel invasion assays

The effects of TIMP-4 overexpression on in vitro invasion were analysed using Matrigel coated Boyden chambers. As seen in Figure 3A, two independent U87 clones transfected with pBabe-TIMP-4 (T4–4 and T4–11) showed approximately 85% and 70% decreased invasion compared to vector control (VC), which was taken as 100% invasion. The T4–4 and T4–11 U87 clones showed approximately 5-fold increased levels of TIMP-4 protein compared to vector control tranfected cells (Figure 3B). TIMP-4 was present in conditioned media of glioma cells and as a cell-associated protein (data not shown). Cell-associated TIMP-4 was primarily cytosolic rather than membrane-associated (Figure 3B). Overexpression of TIMP-4 by the U87 cells had no effect on cell growth rate or morphology (data not shown).

Treatment of glioma cells with exogenous recombinant TIMP-4 gave similar results as observed with the TIMP-4 overexpressing cell lines (Figure 3C). Invasion was decreased by ~75% through Matrigel for U251 cells in the presence of 1 μg $ml^{-1}$ TIMP-4 compared to the control, vehicle-treated cells. This suggests that, whether overexpressed or encountered exogenously by glioma cells, TIMP-4 is a negative regulator of invasive capacity.

### TIMP-4 effects on endothelial cell pro-MMP-2 activation

TIMP-1 is ineffective as an inhibitor of MT-MMPs, though TIMP-2 and TIMP-3 both efficiently block these enzymes (Will et al, 1996; Butler et al, 1997). Sequence comparisons show that TIMP-4 is most closely related to TIMP-2, suggesting that like TIMP-2 it may be an effective inhibitor of MT-MMP activity. Human umbilical vein endothelial cells (HUVEC) secrete pro-MMP-2 (gelatinase-A) and activate the enzyme on the cell surface in response to stimulation by phorbol ester (PMA) or angiogenic factors via the action of MT-MMPs (Foda et al, 1996). As shown in Figure 4, recombinant TIMP-4, like TIMP-2, suppressed PMA-induced pro-MMP-2 activation in HUVECs, whereas TIMP-1 failed to do so. This confirms that TIMP-4 is a functional inhibitor of cell surface MT-MMPs in endothelial cells. HUVECs express very low levels of TIMP-4 transcripts, as determined by quantitative TaqMan RT-PCR (data not shown). Exogenous TIMP-4 also suppressed pro-MMP-2 activation in glioma (U87) and fibrosarcoma cells (HT1080, data not shown).

© 2001 Cancer Research Campaign

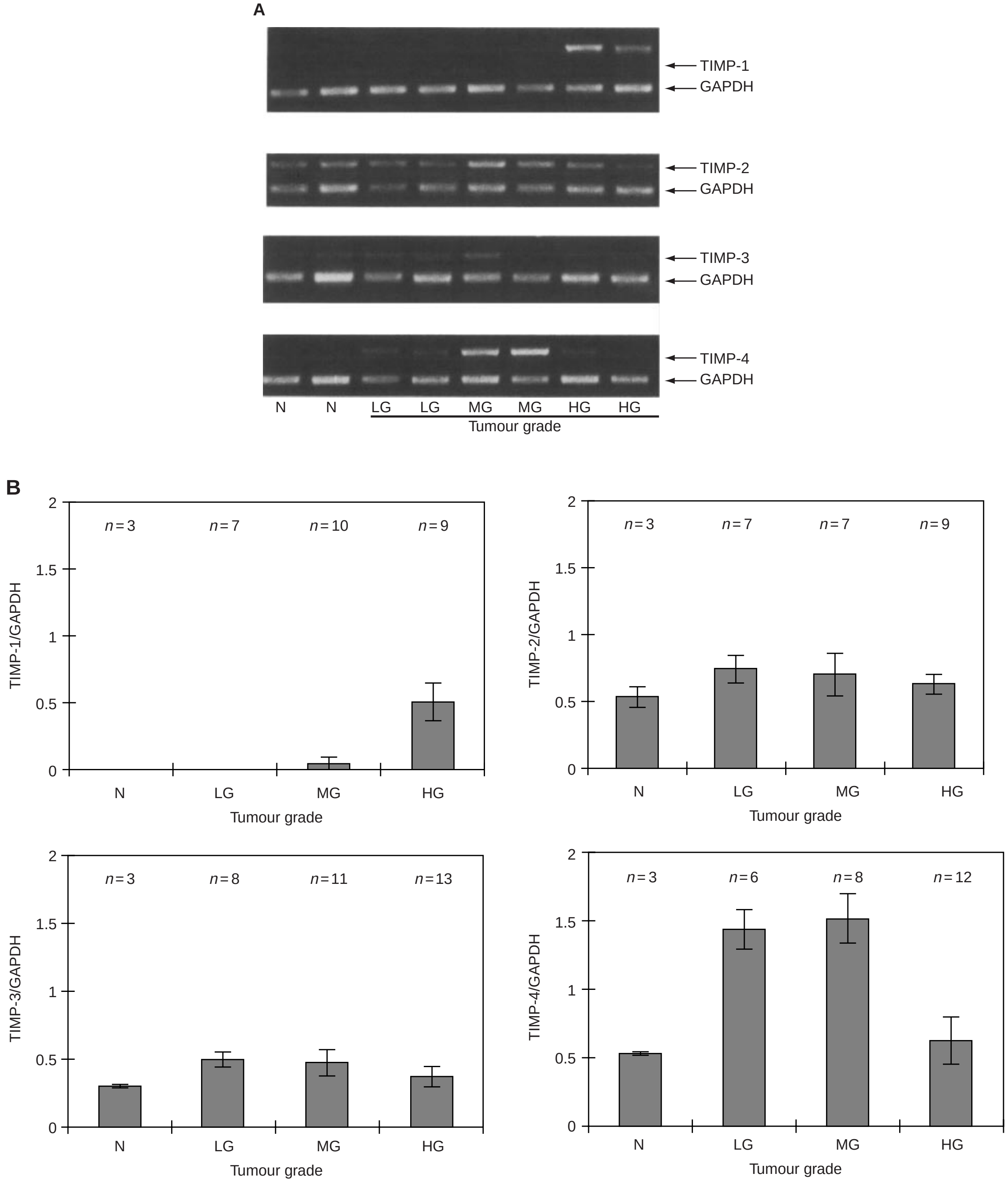

**Figure 1** Quantification of TIMP 1–4 mRNAs in gliomas by RT-PCR. Relative levels for mRNAs for each of the four TIMP genes were compared to the expression of a control gene, GAPDH, in glioma surgical specimens and normal brain tissue using 'primer-dropping' RT-PCR. Panel (**A**) shows a representative gel analysis of PCR products from 2 samples of normal brain tissue and 6 glioma surgical specimens; LG, low grade; MG, middle grade (includes anaplastic astrocytomas and malignant oligodendrogliomas); HG, high grade (glioblastoma multiforme). Panel (**B**) shows the compiled data from the indicated numbers of specimens, with error bars indicating standard deviations; N, normal brain

### DISCUSSION

To the best of our knowledge this is the only report of TIMP-4 in gliomas and the most extensive study of the TIMP family in human gliomas to date. Based on their different patterns of expression and localization, our data suggest that TIMPs-1 and -4, may be involved in the progression of gliomas and that their roles could be distinct. Other studies have addressed the putative link between TIMP expression and brain tumours. Mohanam et al (1995) found TIMP-1 and -2 were down-regulated in GBMs

© 2001 Cancer Research Campaign 

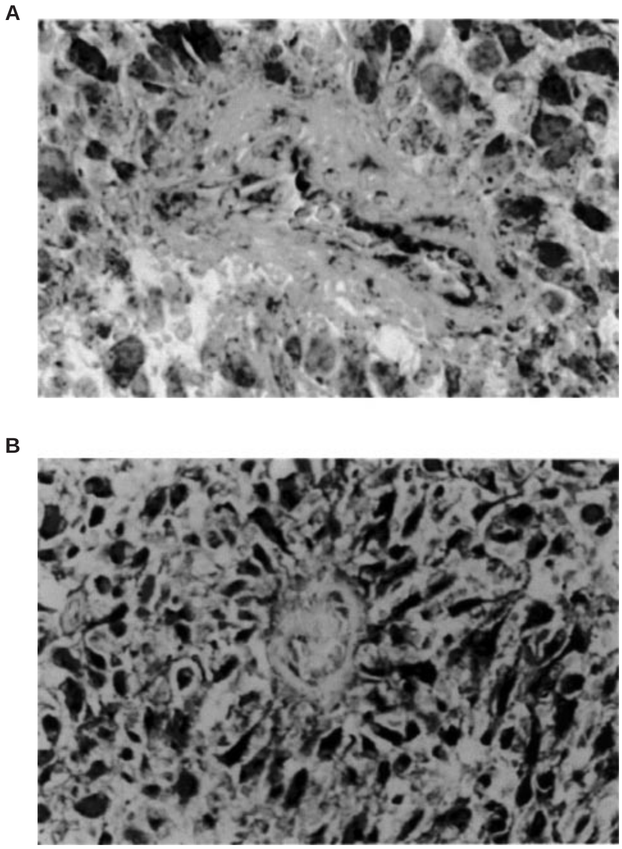

**Figure 2** In situ hybridization analysis of TIMP-1 and TIMP-4 in glioblastoma multiforme. In situ hybridizations were carried out using digoxygenin-labelled riboprobes for TIMP-1 and TIMP-4 on a high grade glioma (glioblastoma multiforme). The TIMP-1 antisense riboprobe (**A**) showed strong hybridization to endothelial cells lining the large blood vessel in the centre of the field and also to surrounding malignant glioma cells. Panel **B** shows results with the TIMP-4 antisense riboprobe on a section from the same tumour, in which hybridization to glioma cells is apparent, but the blood vessel in the centre of the field shows relatively little staining. Magnification 400x in both panels. Sense riboprobe controls showed no signals

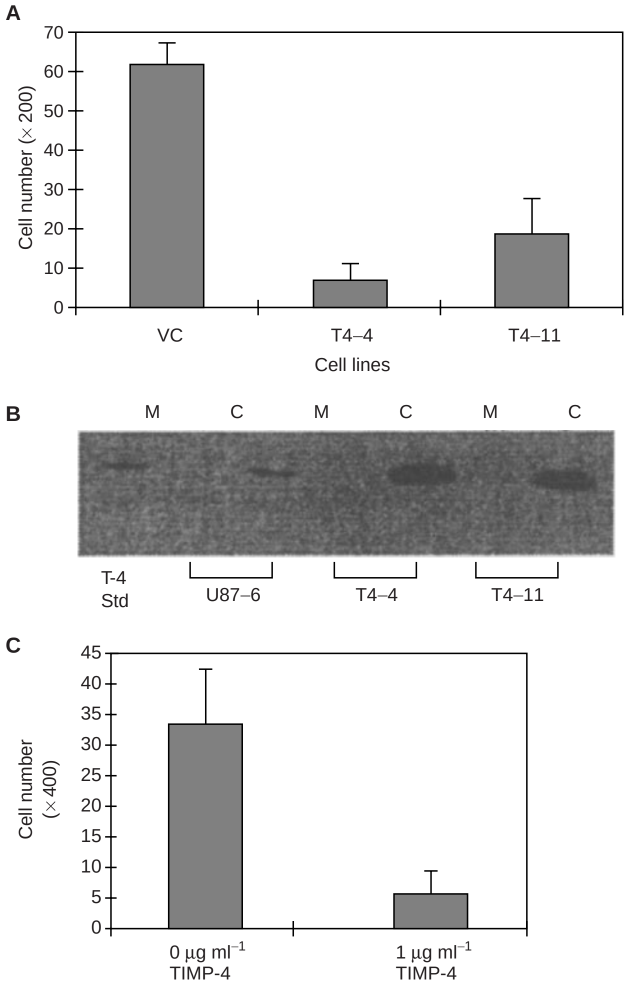

**Figure 3** TIMP-4 inhibits Matrigel invasion of glioma cells. (**A**) Two independent TIMP-4 expressing stably transfected U87 glioma cell lines (T4–4 and T4–11) were compared for the ability to invade Matrigel alongside control pBabe-puro vector control (VC) transfected cells. After 24 hours incubation, cells present on the undersides of Matrigel-coated filters in modified Boyden chambers were fixed, haematoxylin counterstained and counted. Assays were performed in triplicate and 4 random fields at 200x magnification were counted for each assay. (**B**) Western blot analysis of TIMP-4 expression by vector control transfected U87 cells (U87–6) and the T4–4 and T4–11 cell lines. The panel shows results for cell-associated TIMP-4 following cell fractionation (M, membrane-associated; C, cytosolic). Similar results were obtained using conditioned media from the cell lines. (**C**) Matrigel invasion assays were carried out for 36 hours using U251 glioma cells, with either 0 or 1 µg $ml^{-1}$ recombinant murine TIMP-4 (rTIMP-4) added to both the fluid Matrigel prior to solidification and the medium of the upper chamber. Assays were performed in triplicate with four 400× fields counted per assay compared to lower-grade tumours (Mohanam et al, 1995), but we and others (Nakano et al, 1995; Lampert et al, 1998) found TIMP-1 expression increases dramatically with glioma malignancy. Also, TIMP-2 expression does not change with glioma grade (Lampert et al, 1998) though again there is not universal agreement (Mohanam et al, 1995; Saxena et al, 1995). While it may not be relevant in vivo (Rutka et al, 1995) found no correlation between TIMP-1 and -2 expression and glioma cell invasiveness in vitro. As in the Lampert et al (1998) study, we found that TIMP-3 expression did not vary with glioma grade; however, one group found reduced expression by gene silencing via methylation (Bachman et al, 1999). No published reports to date have addressed TIMP-4 expression in human brain tumours.

The precise role of TIMP-1 in glioma pathophysiology is unclear. The increase in TIMP-1 expression seen in many malignant neoplasms may be a general consequence of elevated production or mobilization of growth factors and cytokines in more aggressive tumours, in much the same way that these stimuli lead to induction of MMP-9 or activation of MMP-2. Alternatively, since TIMP-1 preferentially binds the pro-enzyme form of MMP-9, upregulation of this inhibitor in GBM may reflect a specific regulatory mechanism that links these two functions, and could be seen as an attempt by the host to neutralize increased MMP-9 activity and thus slow invasion or angiogenesis. It is also possible that TIMP-1 may stimulate glioma proliferation directly in vivo since TIMP-1 has erythroid potentiating activity (EPA), and stimulates proliferation of numerous cell types (Gasson et al, 1985; Bertaux et al, 1991; Hayakawa et al, 1992, 1994). Moreover, TIMP-1, but not TIMP-2 or the synthetic hydroxamate inhibitor BB-94, has been shown to confer resistance to apoptosis in Burkitt's lymphoma cells (Guedez et al, 1998). Fluorescence-tagged TIMP-1 has been visualized binding to the cell surface of MCF-7 cells, with subsequent translocation to nucleus (Ritter et al, 1999), which may be related to its growth modulatory effects. Finally, though the mechanism is unknown, TIMP-1 may upregulate VEGF expression in gliomas, in

© 2001 Cancer Research Campaign

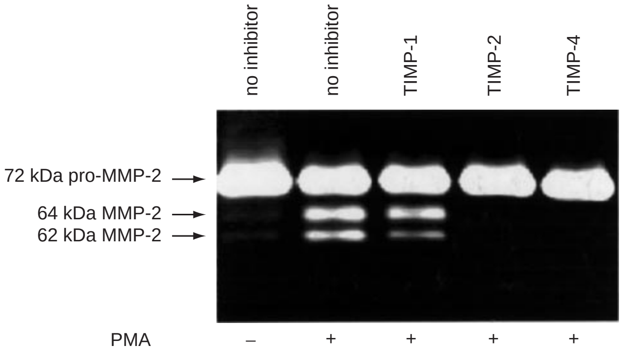

**Figure 4** TIMP-4 inhibits endothelial MT-MMP activity. Human umbilical vein endothelial cells (HUVEC) were grown to confluence and then transferred to serum-free medium in the presence or absence of $10^{-7}$ M PMA and the indicated recombinant TIMPs at 5 µg $ml^{-1}$. After 24 hours, conditioned medium was collected and analysed by gelatin zymography. The position of 72 kDa pro-MMP-2 is indicated, along with that of the MT-MMP-processed 641 kDa intermediate band, and fully activated 62 kDa MMP-2. TIMP-2 and TIMP-4, but not TIMP-1, blocked MT-MMP-mediated activation of pro-MMP-2

parallel with its effects on rat mammary carcinoma cell lines (Yoshiji et al, 1998). Given its potential growth-promoting abilities, TIMP-1 may actually contribute to glioma malignancy when upregulated, rather than act as an inhibitor, though our data do not distinguish between these two possibilities. However, whatever the mechanism, our data suggest that TIMP-1 elevation is a marker of worse clinical outcome in gliomas.

TIMP-4, the newest member of the TIMP family, has not previously been studied in brain tumours. In breast cancer overexpression in vitro and in vivo resulted in decreased invasion, and microvascular density (Wang et al, 1997). One interpretation of our data is that TIMP-4 acts in a similar fashion in gliomas, and its underexpression in highly malignant gliomas allows increased proliferation, invasion and angiogenesis to occur. Consistent with this idea, recombinant or over-expressed TIMP-4 had no effect on cellular viability and proliferation (as measured by $^{3}$H-thymidine incorporation and MTT assays, data not shown), but substantially reduced the invasive capacity of glioma cells through Matrigel. Several MMPs have been linked with the invasive behaviour of malignant gliomas, including MMP-2, MMP-9 and MT1-MMP (Saxena et al, 1995; Sawaya et al, 1996; Uhm et al, 1996; Yamamoto et al, 1996; Deryugina et al, 1998; Lampert et al, 1998; Belien et al, 1999; Forsyth et al, 1999; Price et al, 1999; Raithatha et al, 2000). In particular there is growing evidence for involvement of several other members of the MT-MMP family including MT2-MMP (Nakada et al, 1999), MT5-MMP (Llano et al, 1999) and MT6-MMP (Velasco et al, 2000). Like TIMP-2, TIMP-4 is able to inhibit a wide range of MMPs, including MT-MMPs, as we show by its ability to block pro-MMP-2 activation in HUVECs. Thus, the diminution of TIMP-4 in GBMs compared to lower-grade tumours, combined with increases in a number of MMPs, could contribute to a tilt in the proteolytic balance in favour of increased remodelling and cellular invasion.

The localization data that we have obtained also suggest distinct roles for TIMP-1 and -4 in glioma tumorigenesis. The intense staining in the microvasculature of GBM samples in TIMP-1 in situ hybridization studies is of particular interest. This not only contrasts our localization data of TIMP-4, but also our in situ hybridization data for TIMP-2, where transcript was absent from vascular structures (data not shown). These data are intriguing, as previous reports have suggested that TIMP-2 inhibits cytokine-induced endothelial cell proliferation (Murphy et al, 1993). TIMP-4 overexpression in breast carcinoma cells resulted in less vascular tumours in vivo (Wang et al, 1997). Several pieces of evidence suggest that MT-MMPs are effectors of neovascularization, including the finding that MT1-MMP has been shown to act as a pericellular fibrinolysin and promote neovessel formation in vitro (Hiraoka et al, 1998). Moreover MT1-MMP–/– mice show profound growth abnormalities related to defects in vascularization of the growth plate during osteogenesis, and fail to induce vessel formation in corneal micropocket assays in response to bFGF (Zhou et al, 2000). Therefore, it is possible that reduced TIMP-4 expression in vivo could permit increased MT-MMP activity in the brain microvasculature, leading to increased vessel growth. This idea is supported by in vitro invasion assays, where TIMP-4 inhibited VEGF/bFGF induced formation of 3-dimensional endothelial tube-like structures in fibrin gels (M. Lafleur, unpublished observation). Since TIMP-1 does not inhibit MT-MMPs, its increased presence in endothelial cells in gliomas would have no impact on MT-MMP-mediated angiogenesis.

In summary, our data offer evidence for the differential involvement of TIMPs in glioma pathophysiology. Expression and localization data for mRNA and protein suggest the roles of TIMP-1 and -4 are very different from each other in gliomas though their precise functions are not yet known. Expanding our knowledge of how TIMPs are involved in brain tumour progression will help clarify glioma biology and potentially lead to gene therapy-based approaches of TIMP-delivery to reduce both glioma invasion and angiogenesis.

### ACKNOWLEDGEMENTS

This work was supported by the Medical Research Council of Canada, and the Norfolk and Norwich Big C Appeal. LLG and MAL were the recipients of Natural Sciences and Engineering Research Council of Canada studentships.

### REFERENCES

Ahonen M, Baker AH and Kahari VM (1998) Adenovirus-mediated gene delivery of tissue inhibitor of metalloproteinases-3 inhibits invasion and induces apoptosis in melanoma cells. *Cancer Res* **58**: 2310–2315

Apte S, Olsen BR and Murphy G (1995) The gene structure of tissue inhibitor of metalloproteinases (TIMP)-3 and its inhibitory activities define the distinct TIMP gene family. *J Biol Chem* **270**: 14313–14318

Bachman KE, Herman JG, Corn PG, Merlo A and Costello JF, et al (1999) Methylation-associated silencing of the tissue inhibitor of metalloproteinase-3 gene suggest a suppressor role in kidney, brain, and other human cancers. *Cancer Res* **59**: 798–802

Baker AH, Zaltsman AB, George SJ and Newby AC (1998) Divergent effects of tissue inhibitor of metalloproteinase-1, -2, or-3 overexpression of rat vascular smooth muscle cell invasion, proliferation, and death in vitro. *J Clin Invest* **101**: 1478–1487

Belien AT, Paganetti PA and Schwab ME (1999) Membrane-type 1 matrix metalloproteinase (MT1-MMP) enables invasive migration of glioma cells in central nervous system white matter. *J Cell Biol* **144**: 373–384

Bertaux B, Hornebeck W, Eisen AZ and Dubertret L (1991) Growth stimulation of human keratinocytes by tissue inhibitor of metalloproteinases. *J Invest Dermatol* **97**: 679–685

Blavier L, Henriet P, Imren S and Declerck YA (1999) Tissue inhibitors of matrix metalloproteinases in cancer. *Ann NY Acad Sci* **878**: 108–119

Butler GS, Will H, Atkinson SJ and Murphy G (1997) Membrane-type-2 matrix metalloproteinase can initiate the processing of progelatinase A and is regulated by the tissue inhibitors of metalloproteinases. *Eur J Biochem* **244**: 653–657

*© 2001 Cancer Research Campaign*

Chambers AF and Matrisian LM (1997) Changing views of the role of matrix metalloproteinases in metastasis. *J Natl Cancer Inst* **89**: 1260–1270

DeClerck YA, Yean TD, Chan D, Shimada H and Langely KE (1991) Inhibition of tumour invasion of smooth muscle cell layers by recombinant human metalloproteinase inhibitor. *Cancer Res* **51**: 2151–2157

DeClerck YA, Perez N, Shimada H, Boon TC, Langley KE and Taylor SM (1992) Inhibition of invasion and metastasis in cells transfected with an inhibitor of metalloproteinases. *Cancer Res* **52**: 701–708

Deryugina EI, Bourdon MA, Reisfeld RA and Strongin A (1998) Remodeling of collagen matrix by human tumor cells requires activation and cell surface association of matrix metalloproteinase-2. *Cancer Res* **58**: 3743–3750

Foda HD, George S, Conner C, Drews M, Tompkins DC and Zucker S (1996) Activation of human umbilical vein endothelial cell progelatinase A by phorbol myristate acetate: a protein kinase C-dependent mechanism involving a membrane-type matrix metalloproteinase. *Lab Invest* **74**: 538–545

Forsyth PA, Wong H, Dickson Laing T, Rewcastle NB, Morris DG, et al (1999) Gelatinase-A (MMP-2), gelatinase-B (MMP-9) and membrane type matrix metalloproteinase-1 (MT1-MMP) are involved in different aspects of the pathophysiology of malignant gliomas. *Br J Cancer* **79**: 1828–1835

Gasson JC, Golde DW, Kaufman SE, Westbrook CA and Hewick RM et al (1985) Molecular characterization and expression of the gene encoding human erythroid-potentiating activity. *Nature* **315**: 768–771

Greene J, Wang M, Liu YE, Raymond LA, Rosen C and Shi YE (1996) Molecular cloning and characterization of human tissue inhibitor of metalloproteinase 4. *J Biol Chem* **271**: 30375–30380

Guedez L, Courtemanch L and Stetler-Stevenson M (1998) Tissue inhibitor of metalloproteinase (TIMP)-1 induces differentiation and an antiapoptotic phenotype in germinal center B cells. *Blood* **92**: 1342–1349

Hayakawa T, Yamashita K, Tanzawa K, Uchijima E and Iwata K (1992) Growth-promoting activity of tissue inhibitor of metalloproteinase-1 (TIMP-1) *FEBS Lett* **298**: 29–32

Hayakawa T, Yamashita K, Ohuchi E and Shinnagawa A (1994) Cell growth promoting activity of tissue inhibitor of metalloproteinases-2 (TIMP-2) *J Cell Sci* **107**: 2373–2379

Hiraoka N, Allen E, Apel IJ, Gyetko MR and Weiss SJ (1998) Matrix metalloproteinases regulate neovascularization by acting as pericellular fibrinolysins. *Cell* **95**: 365–377

Holten-Anderson MN, Murphy G, Nielsen HJ, Pedersen AN, Christensen IJ, Hoyer-Hansen G, Brunner N and Stephens RW (1999) Quantitation of TIMP-1 in plasma of healthy blood donors and patients with advanced cancer. *Br J Cancer* **80**: 495–503

Kachra Z, Beaulieu E, Delbecchi L, Mousseau N, Berthelet R, et al (1999) Expression of matrix metalloproteinases and their inhibitors in human brain tumors. *Clin Exp Metastasis* **17**: 555–566

Khokha R, Waterhouse P, Yagel S, Lala PK, Overall CM, et al (1989) Antisense RNA-induced reduction in murine TIMP levels confers oncogenicity on Swiss 3T3 cells. *Science* **243**: 947–950

Kossakowska AE, Urbanski SJ and Edwards DR (1991) Tissue inhibitor of metalloproteinases-1 (TIMP-1) RNA is expressed at elevated levels in malignant non-Hodgkin’s lymphomas. *Blood* **77**: 2475–2481

Lampert K, Machein U, Machein MR, Conca W, Peter HH and Volk B (1998) Expression of matrix metalloproteinases and their tissue inhibitors in human brain tumors. *Am J Pathol* **153**: 429–437

Leco KJ, Khokha R, Pavloff N, Hawkes SP, and Edwards DR (1994) Tissue inhibitor of metalloproteinase-3 (TIMP-3) is an extracellular matrix-associated protein with a distinctive pattern of expression in mouse cells and tissues. *J Biol Chem* **269**: 9352–9360

Leco KJ, Apte SS, Taniguchi GT, Hawkes SP, Khokha R, et al (1997) Murine tissue inhibitor of metalloproteinases-4 (TIMP-4): cDNA isolation and expression in adult mouse tissues. *FEBS Lett* **401**: 213–217

Liotta LA, Steeg PS and Stetler-Stevenson WG (1991) Cancer metastasis and angiogenesis: an imbalance of positive and negative regulation. *Cell* **54**: 327–336

Llano E, Pendas AM, Freije JP, Nakano A, Knauper V, et al (1999) Identification and characterization of human MT5-MMP, a new membrane-bound activator of progelatinase A overexpressed in brain tumors. *Cancer Res* **59**: 2570–2576

Mohanam S, Wang WW, Rayford A, Yamamoto M, Sawaya R, et al (1995) Expression of tissue inhibitors of metalloproteinases: negative regulators of human glioblastoma invasion in vivo. *Clin Exp Metastasis* **13**: 57–62

Murphy A, Unsworth E and Stetler-Stevenson WG (1993) Tissue inhibitor of metalloproteinase-2 (TIMP-2) inhibits bFGF-induced human microvascular endothelial cell proliferation. *J Cell Physiol* **157**: 351–358

Murphy G and Knauper V (1997) Relating matrix metalloproteinase structure to function: why the “hemopexin” domain? *Matrix Biol* **15**: 511–518

Nagase H and Woessner JFJ (1999) Matrix metalloproteinases. *J Biol Chem* **274**: 21491–21494

Nakada M, Nakamura H, Ikeda E, Fujimoto N, Yamashita J, et al (1999) Expression and tissue localization of membrane-type 1, 2, and 3 matrix metalloproteinases in human astrocytic tumors. *Am J Pathol* **154**: 417–428

Nakano A, Tani E, Miuazaki K, Yamamoto U and Furuyama JI (1995) Matrix metalloproteinases and tissue inhibitors of metalloproteinases in human gliomas. *J Neurosurg* **83**: 298–307

Phillips BW, Sharma R, Leco PA and Edwards DR (1999) A sequence-selective single-strand DNA-binding protein regulates basal transcription of the mouse tissue inhibitor of metalloproteinases-1 (TIMP-1) gene. *J Biol Chem* **274**: 22197–22207

Powe DG, Brough JL, Carter GI, Bailey EM, Stetler-Stevenson WG, et al (1997) TIMP-3 mRNA expression is regionally increased in moderately and poorly differentiated colorectal adenocarcinoma. *Br J Cancer* **75**: 1678–1683

Price A, Shi Q, Morris D, Wilcox M, Brasher PM, et al (1999) Marked inhibition of tumor growth in a malignant glioma tumor model by a novel synthetic matrix metalloproteinase inhibitor AG3340. *Cancer Res* **5**: 845–854

Raithatha SA, Muzik H, Rewcastle NB, Johnston RN, Edwards DR and Forsyth PA (2000) Localization of gelatinase-A and gelatinase-B mRNA and protein in human gliomas. *Neuro-Oncol* 3

Ritter LM, Garfield SH and Thorgeirsson UP (1999) Tissue inhibitor of metalloproteinases-1 (TIMP-1) binds to the cell surface and translocates to the nucleus of human MCF-7 breast carcinoma cells. *Biochem Biophys Res Commun* **257**: 494–499

Rutka JT, Matsuzawa K, Hubbard SL, Fukuyama K, Becker LE, Stetler-Stevenson W, Edwards DR and Dirks PB (1995) Expression of TIMP-1, TIMP-2, 72-and 92-kDa type IV collagenase transcripts in human astrocytoma cell lines: Correlation with astrocytoma cell invasiveness. *Int J Oncol* **6**: 877–884

Sawaya RE, Yamamoto M, Gokaslan ZL, Wang SW, Mohanam S, et al (1996) Expression and localization of 72 kDa type IV collagenase (MMP-2) in human malignant gliomas in vivo. *Clin Exp Metastasis* **14**: 35–42

Saxena A, Robertson JT, Kufta C, Stetler-Stevenson WG and Ali IU (1995) Increased expression of gelatinase A and TIMP-2 in primary human glioblastomas. *Int J Oncology* **7**: 469–473

Smith MR, Kung H, Durum SK, Colburn NH and Sun Y (1997) TIMP-3 induces cell death by stabilizing TNF-alpha receptors on the surface of human colon carcinoma cells. *Cytokine* **9**: 770–780

Stetler-Stevenson M, Mansoor A, Lim M, Fukushima P, Kehrl J, et al (1997) Expression of matrix metalloproteinases and tissue inhibitors of metalloproteinases in reactive and neoplastic lymphoid cells. *Blood* **89**: 1708–1715

Uhm JH, Dooley NP, Villemure JG, Yong VW (1996) Glioma invasion in vitro: regulation by matrix metalloprotease-2 and protein kinase C. *Clin Exp Metastasis* **14**: 421–433

Uhm JH, Dooley NP, Villemure JG and Yong VW (1997) Mechanisms of glioma invasion: role of matrix-metalloproteinases. *Canadian Journal of Neurological Science* **24**: 3–15

Velasco G, Cal S, Merlos-Suarez A, Ferrando AA, Alvarez S, et al (2000) Human MT6-matrix metalloproteinase: identification, progelatinase A activation, and expression in brain tumors. *Cancer Res* **60**: 877–882

Wang M, Liu YE, Greene J, Sheng S, Fuchs A ,et al (1997) Inhibition of tumor growth and metastasis of human breast cancer cells transfected with tissue inhibitor of metalloproteinase 4. *Oncogene* **14**: 2767–2774

Will H, Atkinson SJ, Butler GS, Smith B, Murphy G (1996) The soluble catalytic domain of membrane type 1 matrix metalloproteinase cleaves the propetide of progelatinase A and initiates autoproteolytic activation. *J Biol Chem* **271**: 17119–17123

Wingfield PT, Sax JK, Stahl SJ, Kaufman J, Palmer I, Chung V, Corcoran ML, Kleiner DE and Stetler-Stevenson WG (1999). Biophysical and functional characterization of full length, recombinant human tissue inhibitor of metalloproteinases-2 (TIMP-2) produced in *Escherischia coli*. Comparison of wild type and amino-terminal alanine appended variant with implications for the mechanism of TIMP functions. *J Biol Chem* **274**: 21362–21368

Wong H, Anderson WD, Cheng T, Riabowol KT (1994) Monitoring mRNA expression by polymerase chain reaction: the “primer- dropping” method. *Anal Biochem* **223**: 251–258

Yamamoto M, Mohanam S, Sawaya R, Fuller GN, Seiki M, et al (1996) Differential expression of membrane-type matrix metalloproteinase and its correlation with gelatinase A activation in human malignant brain tumors in vivo and in vitro. *Cancer Res* **56**: 384–392

Yong VW, Krekoski CA, Forsyth PA, Bell R, Edwards DR (1998) Matrix metalloproteinases and diseases of the CNS. *Trends Neurosci* **21**: 78–80

 *© 2001 Cancer Research Campaign*

Yoshiji H, Harris SR, Raso E, Gomez DE, Lindsay CK, et al (1998) Mammary carcinoma cells over-expressing tissue inhibitor of metalloproteinases-1 show enhanced vascular endothelial growth factor expression. *Int J Cancer* **75**: 81–87

Zeng ZS, Cohen AM, Zhang ZF, Stetler-Stevenson W, Guillem JG (1995) Elevated tissue inhibitor of metalloproteinases-1 RNA in colorectal cancer stroma correlates with lymph node and distant metastases. *Clin Cancer Res* **1**: 899–906

Zhou Z, Apte SS, Soininen R, Cao R, Baaklini GY, et al (2000) Impaired endochondral ossification and angiogenesis in mice deficient in membrane-type matrix metalloproteinase I. *Proc Natl Acad Sci USA* **97**: 4052– 4057

*© 2001 Cancer Research Campaign* 

## References

[PDF_00678] Ahonen M., Baker A. H., Kähäri V. M. (1998). Adenovirus-mediated gene delivery of tissue inhibitor of metalloproteinases-3 inhibits invasion and induces apoptosis in melanoma cells.. Cancer Res.

[PDF_00681] Apte S. S., Olsen B. R., Murphy G. (1995). The gene structure of tissue inhibitor of metalloproteinases (TIMP)-3 and its inhibitory activities define the distinct TIMP gene family.. J Biol Chem.

[PDF_00684] Bachman K. E., Herman J. G., Corn P. G., Merlo A., Costello J. F., Cavenee W. K., Baylin S. B., Graff J. R. (1999). Methylation-associated silencing of the tissue inhibitor of metalloproteinase-3 gene suggest a suppressor role in kidney, brain, and other human cancers.. Cancer Res.

[PDF_00688] Baker A. H., Zaltsman A. B., George S. J., Newby A. C. (1998). Divergent effects of tissue inhibitor of metalloproteinase-1, -2, or -3 overexpression on rat vascular smooth muscle cell invasion, proliferation, and death in vitro. TIMP-3 promotes apoptosis.. J Clin Invest.

[PDF_00692] Beliën A. T., Paganetti P. A., Schwab M. E. (1999). Membrane-type 1 matrix metalloprotease (MT1-MMP) enables invasive migration of glioma cells in central nervous system white matter.. J Cell Biol.

[PDF_00695] Bertaux B., Hornebeck W., Eisen A. Z., Dubertret L. (1991). Growth stimulation of human keratinocytes by tissue inhibitor of metalloproteinases.. J Invest Dermatol.

[PDF_00698] Blavier L., Henriet P., Imren S., Declerck Y. A. (1999). Tissue inhibitors of matrix metalloproteinases in cancer.. Ann N Y Acad Sci.

[PDF_00700] Butler G. S., Will H., Atkinson S. J., Murphy G. (1997). Membrane-type-2 matrix metalloproteinase can initiate the processing of progelatinase A and is regulated by the tissue inhibitors of metalloproteinases.. Eur J Biochem.

[PDF_00725] Chambers A. F., Matrisian L. M. (1997). Changing views of the role of matrix metalloproteinases in metastasis.. J Natl Cancer Inst.

[PDF_00730] DeClerck Y. A., Perez N., Shimada H., Boone T. C., Langley K. E., Taylor S. M. (1992). Inhibition of invasion and metastasis in cells transfected with an inhibitor of metalloproteinases.. Cancer Res.

[PDF_00727] DeClerck Y. A., Yean T. D., Chan D., Shimada H., Langley K. E. (1991). Inhibition of tumor invasion of smooth muscle cell layers by recombinant human metalloproteinase inhibitor.. Cancer Res.

[PDF_00733] Deryugina E. I., Bourdon M. A., Reisfeld R. A., Strongin A. (1998). Remodeling of collagen matrix by human tumor cells requires activation and cell surface association of matrix metalloproteinase-2.. Cancer Res.

[PDF_00736] Foda H. D., George S., Conner C., Drews M., Tompkins D. C., Zucker S. (1996). Activation of human umbilical vein endothelial cell progelatinase A by phorbol myristate acetate: a protein kinase C-dependent mechanism involving a membrane-type matrix metalloproteinase.. Lab Invest.

[PDF_00740] Forsyth P. A., Wong H., Laing T. D., Rewcastle N. B., Morris D. G., Muzik H., Leco K. J., Johnston R. N., Brasher P. M., Sutherland G. (1999). Gelatinase-A (MMP-2), gelatinase-B (MMP-9) and membrane type matrix metalloproteinase-1 (MT1-MMP) are involved in different aspects of the pathophysiology of malignant gliomas.. Br J Cancer.

[PDF_00744] Gasson J. C., Golde D. W., Kaufman S. E., Westbrook C. A., Hewick R. M., Kaufman R. J., Wong G. G., Temple P. A., Leary A. C., Brown E. L. Molecular characterization and expression of the gene encoding human erythroid-potentiating activity.. Nature.

[PDF_00747] Greene J., Wang M., Liu Y. E., Raymond L. A., Rosen C., Shi Y. E. (1996). Molecular cloning and characterization of human tissue inhibitor of metalloproteinase 4.. J Biol Chem.

[PDF_00750] Guedez L., Courtemanch L., Stetler-Stevenson M. (1998). Tissue inhibitor of metalloproteinase (TIMP)-1 induces differentiation and an antiapoptotic phenotype in germinal center B cells.. Blood.

[PDF_00756] Hayakawa T., Yamashita K., Ohuchi E., Shinagawa A. (1994). Cell growth-promoting activity of tissue inhibitor of metalloproteinases-2 (TIMP-2).. J Cell Sci.

[PDF_00753] Hayakawa T., Yamashita K., Tanzawa K., Uchijima E., Iwata K. (1992). Growth-promoting activity of tissue inhibitor of metalloproteinases-1 (TIMP-1) for a wide range of cells. A possible new growth factor in serum.. FEBS Lett.

[PDF_00759] Hiraoka N., Allen E., Apel I. J., Gyetko M. R., Weiss S. J. (1998). Matrix metalloproteinases regulate neovascularization by acting as pericellular fibrinolysins.. Cell.

[PDF_00763] Holten-Andersen M. N., Murphy G., Nielsen H. J., Pedersen A. N., Christensen I. J., Høyer-Hansen G., Brünner N., Stephens R. W. (1999). Quantitation of TIMP-1 in plasma of healthy blood donors and patients with advanced cancer.. Br J Cancer.

[PDF_00766] Kachra Z., Beaulieu E., Delbecchi L., Mousseau N., Berthelet F., Moumdjian R., Del Maestro R., Béliveau R. (1999). Expression of matrix metalloproteinases and their inhibitors in human brain tumors.. Clin Exp Metastasis.

[PDF_00769] Khokha R., Waterhouse P., Yagel S., Lala P. K., Overall C. M., Norton G., Denhardt D. T. (1989). Antisense RNA-induced reduction in murine TIMP levels confers oncogenicity on Swiss 3T3 cells.. Science.

[PDF_00772] Kossakowska A. E., Urbanski S. J., Edwards D. R. (1991). Tissue inhibitor of metalloproteinases-1 (TIMP-1) RNA is expressed at elevated levels in malignant non-Hodgkin's lymphomas.. Blood.

[PDF_00775] Lampert K., Machein U., Machein M. R., Conca W., Peter H. H., Volk B. (1998). Expression of matrix metalloproteinases and their tissue inhibitors in human brain tumors.. Am J Pathol.

[PDF_00782] Leco K. J., Apte S. S., Taniguchi G. T., Hawkes S. P., Khokha R., Schultz G. A., Edwards D. R. (1997). Murine tissue inhibitor of metalloproteinases-4 (Timp-4): cDNA isolation and expression in adult mouse tissues.. FEBS Lett.

[PDF_00778] Leco K. J., Khokha R., Pavloff N., Hawkes S. P., Edwards D. R. (1994). Tissue inhibitor of metalloproteinases-3 (TIMP-3) is an extracellular matrix-associated protein with a distinctive pattern of expression in mouse cells and tissues.. J Biol Chem.

[PDF_00785] Liotta L. A., Steeg P. S., Stetler-Stevenson W. G. (1991). Cancer metastasis and angiogenesis: an imbalance of positive and negative regulation.. Cell.

[PDF_00788] Llano E., Pendás A. M., Freije J. P., Nakano A., Knäuper V., Murphy G., López-Otin C. (1999). Identification and characterization of human MT5-MMP, a new membrane-bound activator of progelatinase a overexpressed in brain tumors.. Cancer Res.

[PDF_00791] Mohanam S., Wang S. W., Rayford A., Yamamoto M., Sawaya R., Nakajima M., Liotta L. A., Nicolson G. L., Stetler-Stevenson W. G., Rao J. S. (1995). Expression of tissue inhibitors of metalloproteinases: negative regulators of human glioblastoma invasion in vivo.. Clin Exp Metastasis.

[PDF_00794] Murphy A. N., Unsworth E. J., Stetler-Stevenson W. G. (1993). Tissue inhibitor of metalloproteinases-2 inhibits bFGF-induced human microvascular endothelial cell proliferation.. J Cell Physiol.

[PDF_00797] Murphy G., Knäuper V. (1997). Relating matrix metalloproteinase structure to function: why the "hemopexin" domain?. Matrix Biol.

[PDF_00799] Nagase H., Woessner J. F. (1999). Matrix metalloproteinases.. J Biol Chem.

[PDF_00801] Nakada M., Nakamura H., Ikeda E., Fujimoto N., Yamashita J., Sato H., Seiki M., Okada Y. (1999). Expression and tissue localization of membrane-type 1, 2, and 3 matrix metalloproteinases in human astrocytic tumors.. Am J Pathol.

[PDF_00804] Nakano A., Tani E., Miyazaki K., Yamamoto Y., Furuyama J. (1995). Matrix metalloproteinases and tissue inhibitors of metalloproteinases in human gliomas.. J Neurosurg.

[PDF_00807] Phillips B. W., Sharma R., Leco P. A., Edwards D. R. (1999). A sequence-selective single-strand DNA-binding protein regulates basal transcription of the murine tissue inhibitor of metalloproteinases-1 (Timp-1) gene.. J Biol Chem.

[PDF_00811] Powe D. G., Brough J. L., Carter G. I., Bailey E. M., Stetler-Stevenson W. G., Turner D. R., Hewitt R. E. (1997). TIMP-3 mRNA expression is regionally increased in moderately and poorly differentiated colorectal adenocarcinoma.. Br J Cancer.

[PDF_00814] Price A., Shi Q., Morris D., Wilcox M. E., Brasher P. M., Rewcastle N. B., Shalinsky D., Zou H., Appelt K., Johnston R. N. (1999). Marked inhibition of tumor growth in a malignant glioma tumor model by a novel synthetic matrix metalloproteinase inhibitor AG3340.. Clin Cancer Res.

[PDF_00829] Sawaya R. E., Yamamoto M., Gokaslan Z. L., Wang S. W., Mohanam S., Fuller G. N., McCutcheon I. E., Stetler-Stevenson W. G., Nicolson G. L., Rao J. S. (1996). Expression and localization of 72 kDa type IV collagenase (MMP-2) in human malignant gliomas in vivo.. Clin Exp Metastasis.

[PDF_00835] Smith M. R., Kung H., Durum S. K., Colburn N. H., Sun Y. (1997). TIMP-3 induces cell death by stabilizing TNF-alpha receptors on the surface of human colon carcinoma cells.. Cytokine.

[PDF_00838] Stetler-Stevenson M., Mansoor A., Lim M., Fukushima P., Kehrl J., Marti G., Ptaszynski K., Wang J., Stetler-Stevenson W. G. (1997). Expression of matrix metalloproteinases and tissue inhibitors of metalloproteinases in reactive and neoplastic lymphoid cells.. Blood.

[PDF_00841] Uhm J. H., Dooley N. P., Villemure J. G., Yong V. W. (1996). Glioma invasion in vitro: regulation by matrix metalloprotease-2 and protein kinase C.. Clin Exp Metastasis.

[PDF_00844] Uhm J. H., Dooley N. P., Villemure J. G., Yong V. W. (1997). Mechanisms of glioma invasion: role of matrix-metalloproteinases.. Can J Neurol Sci.

[PDF_00847] Velasco G., Cal S., Merlos-Suárez A., Ferrando A. A., Alvarez S., Nakano A., Arribas J., López-Otín C. (2000). Human MT6-matrix metalloproteinase: identification, progelatinase A activation, and expression in brain tumors.. Cancer Res.

[PDF_00850] Wang M., Liu Y. E., Greene J., Sheng S., Fuchs A., Rosen E. M., Shi Y. E. (1997). Inhibition of tumor growth and metastasis of human breast cancer cells transfected with tissue inhibitor of metalloproteinase 4.. Oncogene.

[PDF_00853] Will H., Atkinson S. J., Butler G. S., Smith B., Murphy G. (1996). The soluble catalytic domain of membrane type 1 matrix metalloproteinase cleaves the propeptide of progelatinase A and initiates autoproteolytic activation. Regulation by TIMP-2 and TIMP-3.. J Biol Chem.

[PDF_00857] Wingfield P. T., Sax J. K., Stahl S. J., Kaufman J., Palmer I., Chung V., Corcoran M. L., Kleiner D. E., Stetler-Stevenson W. G. (1999). Biophysical and functional characterization of full-length, recombinant human tissue inhibitor of metalloproteinases-2 (TIMP-2) produced in Escherichia coli. Comparison of wild type and amino-terminal alanine appended variant with implications for the mechanism of TIMP functions.. J Biol Chem.

[PDF_00864] Wong H., Anderson W. D., Cheng T., Riabowol K. T. (1994). Monitoring mRNA expression by polymerase chain reaction: the "primer-dropping" method.. Anal Biochem.

[PDF_00867] Yamamoto M., Mohanam S., Sawaya R., Fuller G. N., Seiki M., Sato H., Gokaslan Z. L., Liotta L. A., Nicolson G. L., Rao J. S. (1996). Differential expression of membrane-type matrix metalloproteinase and its correlation with gelatinase A activation in human malignant brain tumors in vivo and in vitro.. Cancer Res.

[PDF_00871] Yong V. W., Krekoski C. A., Forsyth P. A., Bell R., Edwards D. R. (1998). Matrix metalloproteinases and diseases of the CNS.. Trends Neurosci.

[PDF_00875] Yoshiji H., Harris S. R., Raso E., Gomez D. E., Lindsay C. K., Shibuya M., Sinha C. C., Thorgeirsson U. P. (1998). Mammary carcinoma cells over-expressing tissue inhibitor of metalloproteinases-1 show enhanced vascular endothelial growth factor expression.. Int J Cancer.

[PDF_00879] Zeng Z. S., Cohen A. M., Zhang Z. F., Stetler-Stevenson W., Guillem J. G. (1995). Elevated tissue inhibitor of metalloproteinase 1 RNA in colorectal cancer stroma correlates with lymph node and distant metastases.. Clin Cancer Res.

[PDF_00883] Zhou Z., Apte S. S., Soininen R., Cao R., Baaklini G. Y., Rauser R. W., Wang J., Cao Y., Tryggvason K. (2000). Impaired endochondral ossification and angiogenesis in mice deficient in membrane-type matrix metalloproteinase I.. Proc Natl Acad Sci U S A.

